# Supplementary material for: Antibacterial and Oxidative Stress-Protective Effects of Five Monoterpenes from Softwood
Source: Molecules. 2022 Jun 17;27(12):3891. doi: 10.3390/molecules27123891 (PMC9230896; doi:10.3390/molecules27123891)
Supplement: Supplementary file 1 [file molecules-27-03891-s001.zip › molecules-1741029-supplementary.pdf]

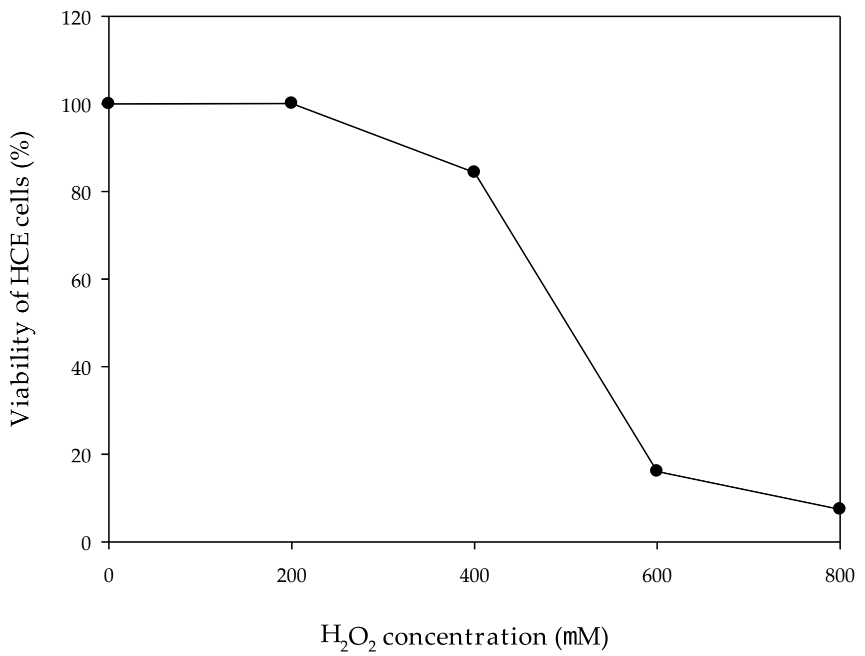

Supplementary Figure S1. Effect of hydrogen peroxide (H_2O_2) on HCE cell viability. Content of 800 μM H_2O_2 killed the cells and was selected for further studies.

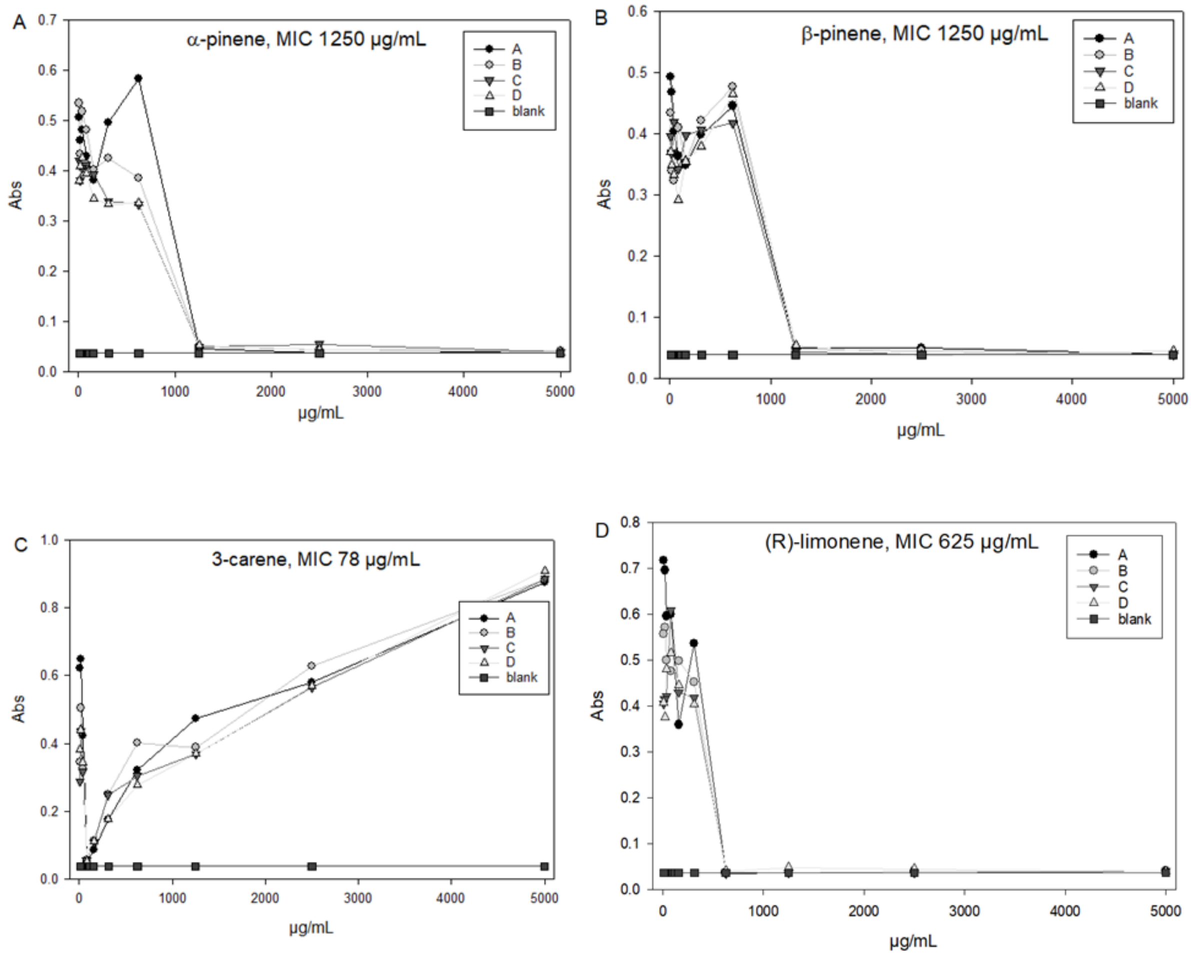

Supplementary Figure S2. MIC test indicated that for α-pinene, β-pinene, 3-carene and R-limonene the bacterial growth inhibiting contents were > 1.25 mg/mL, > 1.25 mg/mL, > 0.078 mg/mL, > 0.63 mg/mL against *E. coli*, respectively.

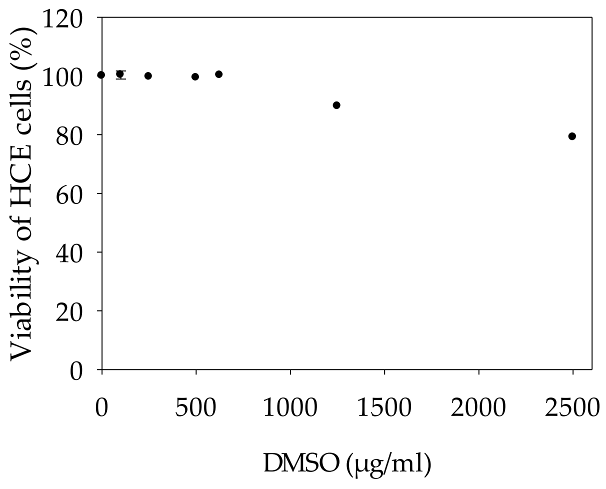

Supplementary Figure S3. Effect of DMSO on HCE cell viability: Terpenes were dissolved in dimethyl sulfoxide (DMSO), which is cytotoxic in high concentrations. The effect of DMSO on HCE cells was tested using terpene dilutions' DMSO concentrations. Lower than 1000 µg/mL DMSO at terpene dilutions had no effect on cell viability.

Supplementary Table S1. Comparisons between the Oxygen Radical Absorbance Capacity (ORAC) of five terpenes analyzed by one-way variance analysis (ANOVA) and Tukey´s multiple comparisons of mean. ORAC-values of 3-carene and S-limonene were on higher level compared to α- and β-pinenes and R-limonene.

Terpene pair	Estimate	Std.Error	t-value	Pr(>|t|)
β-pinene - α-pinene	936.6	4773.6	0.196	1.000
3-carene – α-pinene	38438.6	4465.3	8.608	<1e-04 ***
S-limonene – α-pinene	48671.1	4773.6	10.196	<1e-04 ***
R-limonene – α-pinene	6996.8	4465.3	1.567	0.542
3-carene – β-pinene	37502.0	4465.3	8.399	<1e-04 ***
S-limonene – β-pinene	47734.5	4773.6	10.000	<1e-04 ***
R-limonene – β-pinene	6060.2	4465.3	1.357	0.663
S-limonene – 3-carene	10232.5	4465.3	2.292	0.212
R-limonene – 3-carene	-31441.8	4134.0	-7.606	<1e-04 ***
R-limonene – S-limonene	-41674.3	4465.3	-9.333	<1e-04 ***

Signif. codes: 0 '***' 0.001 '**' 0.01 '*' 0.05 '.' 0.1 ' ' 1

Supplemntary Table S2. Comparisons between the effects of three different α-pinene concentrations, 6.3% DMSO and water treatment on light production of *E. coli* K12+pcGLS11 strain after 50 min incubation analyzed by one-way variance analysis (ANOVA) and Tukey´s multiple mean comparisons. After 50min incubation time there was a difference in light production between all treatments.

α-pinene (mg/ml)	Estimate (RLU)	Std.Error	t-value	Pr(>|t|)
1.6 – 3.2	48036600	1068208	44.969	< 1e-04 ***
0.8 – 3.2	57998500	1068208	54.295	< 1e-04 ***
dmso – 3.2	39609667	1068208	37.080	< 1e-04 ***
water – 3.2	67671367	1068208	63.350	< 1e-04 ***
0.8 – 1.6	9961900	1068208	9.326	< 1e-04 ***
dmso – 1.6	-8426933	1068208	-7.889	0.000113 ***
water – 1.6	19634767	1068208	18.381	< 1e-04 ***
dmso – 0.8	-18388833	1068208	-17.215	< 1e-04 ***
water – 0.8	9672867	1068208	9.055	< 1e-04 ***

Signif. codes: 0 '***' 0.001 '**' 0.01 '*' 0.05 '.' 0.1 ' ' 1

Supplementary Table S3. Comparisons between the effects of three different β-pinene concentrations, 6.3% DMSO and water treatment on light production of *E. coli* K12+pcGLS11 strain after 50 min incubation analyzed by one-way variance analysis (ANOVA) and Tukey´s multiple mean comparisons. Contents 0.8 and 1.6 mg/ml of β-pinene did not vary from each other's and 3.2 mg/ml content was as effective as DMSO treatment.

β-pinene (mg/ml)	Estimate (RLU)	Std.Error	t-value	Pr(>|t|)
1.6 – 3.2	21188767	1822588	11.626	< 1e-04 ***
0.8 – 3.2	20446167	1822588	11.218	< 1e-04 ***
dmso – 3.2	4631333	1822588	2.541	0.156699
water – 3.2	32693033	1822588	17.938	< 1e-04 ***
0.8 – 1.6	-742600	1822588	-0.407	0.993268
dmso – 1.6	-16557433	1822588	-9.085	< 1e-04 ***
water – 1.6	11504267	1822588	6.312	0.000574 ***
dmso – 0.8	-15814833	1822588	-8.677	< 1e-04 ***
water – 0.8	12246867	1822588	6.719	0.000369 ***

Signif. codes: 0 '***' 0.001 '**' 0.01 '*' 0.05 '.' 0.1 ' ' 1

Supplementary Table S4. Comparisons between the effects of three different 3-Carene concentrations, 6.3% DMSO and water treatment on light production of *E. coli* K12+pcGLS11 strain after 50 min incubation analyzed by one-way variance analysis (ANOVA) and Tukey´s multiple mean comparisons. The three 3-carene concentrations did not vary from each other´s after 50 min incubation.

3-carene (mg/ml)	Estimate (RLU)	Std.Error	t-value	Pr(>|t|)
1.6 – 3.2	-88813	392803	-0.226	0.999
0.8 – 3.2	-45051	392803	-0.115	1.000
dmso – 3.2	62749968	392803	159.749	<1e-06 ***
water – 3.2	90811668	392803	231.189	<1e-06 ***
0.8 – 1.6	43762	392803	0.111	1.000
dmso – 1.6	62838781	392803	159.975	<1e-06 ***
water – 1.6	90900481	392803	231.415	<1e-06 ***
dmso – 0.8	62795019	392803	159.864	<1e-06 ***
water – 0.8	90856719	392803	231.304	<1e-06 ***

Signif. codes: 0 '***' 0.001 '**' 0.01 '*' 0.05 '.' 0.1 ' ' 1

Supplementary Table S5. Comparisons between the effects of three different S-limonene concentrations, 6.3% DMSO and water treatment on light production of *E. coli* K12+pcGLS11 strain after 50 min incubation analyzed by one-way variance analysis (ANOVA) and Tukey´s multiple mean comparisons. After 50 min of incubation, there were no differences in light production between the three S-limonene concentration treatments.

S-limonene (mg/ml)	Estimate (RLU)	Std.Error	t-value	Pr(>|t|)
1.6 – 3.2	1320	393211	0.003	1.000
0.8 – 3.2	244585	393211	0.622	0.968
dmso – 3.2	62847733	393211	159.832	<1e-04 ***
water – 3.2	90909433	393211	231.198	<1e-04 ***
0.8 – 1.6	243266	393211	0.619	0.969
dmso – 1.6	62846413	393211	159.829	<1e-04 ***
water – 1.6	90908113	393211	231.194	<1e-04 ***
dmso – 0.8	62603148	393211	159.210	<1e-04 ***
water – 0.8	90664848	393211	230.576	<1e-04 ***

Signif. codes: 0 '***' 0.001 '**' 0.01 '*' 0.05 '.' 0.1 ' ' 1

Supplementary Table S6. Comparisons between the effects of three different α-pinene concentrations, 6.3% DMSO and water treatment on light production of *S. aureus* RN4220+pAT19 strain after 50 min incubation analyzed by one-way variance analysis (ANOVA) and Tukey´s multiple mean comparisons. After 50min incubation time there was a difference in light production between all treatments.

α-pinene (mg/ml)	Estimate (RLU)	Std.Error	t-value	Pr(>|t|)
1.6 – 3.2	22576	1183	19.083	<0.001 ***
0.8 – 3.2	51981	1183	43.938	<0.001 ***
dmso – 3.2	56125	1183	47.441	<0.001 ***
water – 3.2	101009	1183	85.380	<0.001 ***
0.8 – 1.6	29406	1183	24.856	<0.001 ***
dmso – 1.6	33549	1183	28.358	<0.001 ***
water – 1.6	78434	1183	66.298	<0.001 ***
dmso – 0.8	4144	1183	3.503	0.036 *
water – 0.8	49028	1183	41.442	<0.001 ***

Signif. codes: 0 '***' 0.001 '**' 0.01 '*' 0.05 '.' 0.1 ' ' 1

Supplementary Table S7. Comparisons between the effects of three different β-pinene concentrations, 6.3% DMSO and water treatment on light production of *S. aureus* RN4220+pAT19 strain after 50 min incubation analyzed by one-way variance analysis (ANOVA) and Tukey´s multiple mean comparisons. After 50min incubation time, the β-pinene concentration 3.2 mg/ml inhibited light production more than the two other concentrations (1.6mg/ml and 0.8 mg/ml) and DMSO.

β-pinene (mg/ml)	Estimate (RLU)	Std.Error	t-value	Pr(>|t|)
1.6 – 3.2	32360	2300	14.069	< 0.001 ***
0.8 – 3.2	36625	2300	15.924	< 0.001 ***
dmso – 3.2	25616	2300	11.137	< 0.001 ***
water – 3.2	70501	2300	30.652	< 0.001 ***
0.8 – 1.6	4266	2300	1.855	0.39725
dmso – 1.6	-6743	2300	-2.932	0.08707 .
water – 1.6	38141	2300	16.583	< 0.001 ***
dmso – 0.8	-11009	2300	-4.786	0.00516 **
water – 0.8	32360	2300	14.069	< 0.001 ***

Signif. codes: 0 '***' 0.001 '**' 0.01 '*' 0.05 '.' 0.1 ' ' 1

Supplementary Table S8. Comparisons between the effects of three different 3-carene concentrations, 6.3% DMSO and water treatment on light production of *S. aureus* RN4220+pAT19 strain after 50 min incubation analyzed by one-way variance analysis (ANOVA) and Tukey´s multiple mean comparisons. After 50min incubation time, all three 3-carene treatments had similar effect on light production.

3-carene (mg/ml)	Estimate	Std.Error	t-value	Pr(>|t|)
1.6 – 3.2	-337.5	418.5	-0.806	0.923
0.8 – 3.2	-449.2	418.5	-1.073	0.816
dmso – 3.2	57977.5	418.5	138.535	<1e-04 ***
water – 3.2	102861.7	418.5	245.783	<1e-04 ***
0.8 – 1.6	-111.8	418.5	-0.267	0.999
dmso – 1.6	58314.9	418.5	139.341	<1e-04 ***
water – 1.6	103199.1	418.5	246.589	<1e-04 ***
dmso – 0.8	58426.7	418.5	139.608	<1e-04 ***
water – 0.8	103310.9	418.5	246.857	<1e-04 ***

Signif. codes: 0 ‘***’ 0.001 ‘**’ 0.01 ‘*’ 0.05 ‘.’ 0.1 ‘ ’ 1

Supplementary Table S9. Comparisons between the effects of three different S-limonene concentrations, 6.3% DMSO and water treatment on light production of *S. aureus* RN4220+pAT19 strain after 50 min incubation analyzed by one-way variance analysis (ANOVA) and Tukey´s multiple mean comparisons. After 50min incubation time, all three S-limonene treatments had similar effect on light production.

S-limonene (mg/ml)	Estimate	Std.Error	t-value	Pr(>|t|)
1.6 – 3.2	-9.878	418.497	-0.024	1
0.8 – 3.2	6.528	418.497	0.016	1
dmso – 3.2	58499.176	418.497	139.784	<1e-09 ***
water – 3.2	103383.342	418.497	247.035	<1e-09 ***
0.8 – 1.6	16.407	418.497	0.039	1
dmso – 1.6	58509.054	418.497	139.808	<1e-09 ***
water – 1.6	103393.221	418.497	247.059	<1e-09 ***
dmso – 0.8	58492.648	418.497	139.768	<1e-09 ***
water – 0.8	103376.814	418.497	247.019	<1e-09 ***

Signif. codes: 0 ‘***’ 0.001 ‘**’ 0.01 ‘*’ 0.05 ‘.’ 0.1 ‘ ’ 1
